# Delayed Diagnosis of Congenital Duodenal Stenosis in a 16-Year-Old Girl

**DOI:** 10.1155/2024/1070253

**Published:** 2024-05-04

**Authors:** Virtut Velmishi, Dritan Alushani, Ermira Dervishi, Saimir Heta, Spiro Sila, Paskal Cullufi

**Affiliations:** ^1^Service of Pediatric Gastroenterology “Mother Teresa” University Hospital Center, Tirana, Albania; ^2^Service of pediatric Surgery “Mother Teresa” University Hospital Center, Tirana, Albania

## Abstract

**Background:**

Duodenal atresia or stenosis are different degrees of the same abnormality. They usually occur at the level of the ampulla of Vater and are thought to be an embryologic defect during the development of the foregut, leading to abnormal recanalization. Complete duodenal atresia is usually symptomatic in the early neonatal period, while partial obstruction (web, stenosis) may have a late presentation and a more challenging diagnosis such as in our case. *Case Presentation*. The patient, a 16-year-old girl, presented with abdominal pain, recurrent vomiting, and growth failure. An upper GI study with barium showed an image compatible with gastroptosis. Further diagnostic procedures confirmed a rare finding such as congenital duodenal stenosis. She underwent surgical intervention, and the recovery period was uneventful.

**Conclusion:**

Gastroptosis is not diagnostic for a particular disease. This rare radiological finding in children may obscure uncommon diagnosis, such as congenital duodenal stenosis, which can present a diagnostic challenge beyond early childhood.

## 1. Background

Congenital duodenal obstruction caused by duodenal atresia or duodenal stenosis is a rare congenital anomaly. The prevalence of duodenal obstruction (atresia, web, stenosis) is 1 : 6000 [1–3]. Nowadays, approximately half of all cases are detected antenatally on fetal ultrasonography with the characteristic presence of a “double bubble” sign in the upper abdomen representing dilated fluid-filled stomach and proximal duodenum. Suspicion of congenital duodenal obstruction demands an accurate investigation of associated anomalies, such as cardiac and aneuploidy, commonly occurring in up to 84% of infants, depending on the reported population [4]. Congenital duodenal obstruction can occur in association with an annular pancreas, resulting in a partial or complete duodenal obstruction. A significant number of affected infants also have Down syndrome. The definitive management of congenital duodenal stenosis is surgical, aiming to restore gastrointestinal continuity while avoiding damage to adjacent structures, principally the biliary and pancreatic ducts. Surgeons use several different procedures, techniques, and approaches, and none have proven benefit over any other [5, 6]. Most patients with atresia or duodenal stenosis are diagnosed postnatally or at a young age. This case shows a delayed diagnosis of duodenal stenosis in a 16-year-old girl. This manuscript was prepared following the CARE guidelines (https://www.care-statement.org).

## 2. Case Report

A 16-year-old girl was sent to the gastroenterology service because of abdominal pain, recurrent vomiting, and malnutrition. Gastrointestinal symptoms began when she was a little girl. Her parents were divorced and her appointments at healthcare centers were not regular. According to her sister she was never hospitalized but was treated for her gastrointestinal symptoms by her family doctor without significant improvement. No family diseases were reported.

**Figure 1:**
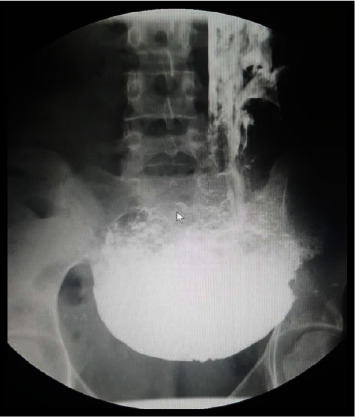
Barium swallow shows gastroptosis: stomach is under the level of iliac crests.

**Figure 2:**
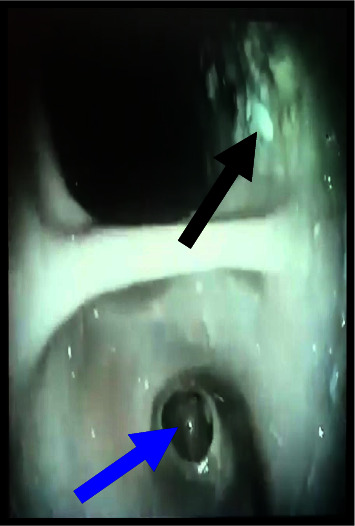
Upper digestive endoscopy shows a narrow duodenal bulb (blue arrow) and some food residues into the stomach (black arrow).

**Figure 3:**
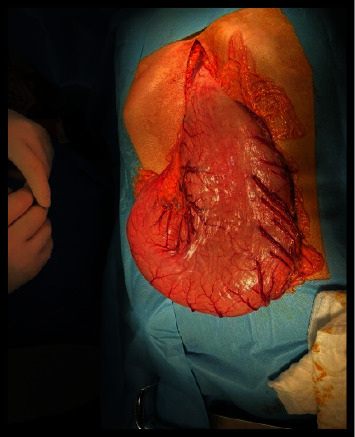
Enlarged stomach during the intervention.

**Figure 4:**
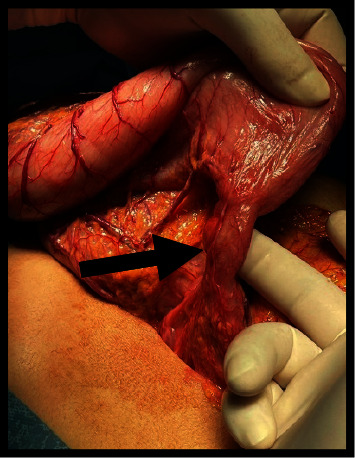
Congenital duodenal stenosis during the intervention.

**Figure 5:**
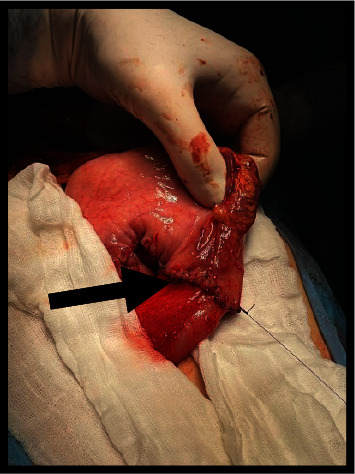
The anastomosis similar to Billroth type 1.

**Figure 6:**
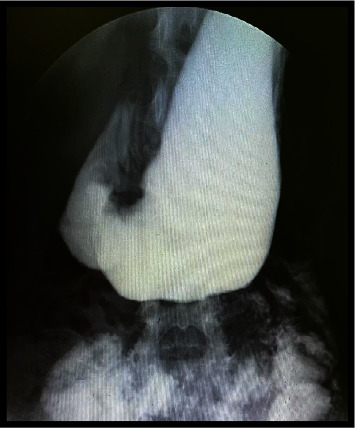
The stomach is in a normal position 3 months after the intervention.

The CBC showed mild anemia, while biochemical examinations were within the normal range. Her objective assessment showed low staturoponderal growth. Her weight was 40 kg (*z* score = −2.30; percentile 1.072%), and her height was 155 cm (*z* score: −1.17; percentile 12%). She was pale, but no facial dysmorphia was noticed. Heart sounds were normal. Her arterial pressure was in the normal range according to her age. Lungs were clear during auscultation. The abdomen was soft without enlargement of the liver or spleen. Her extremities were thin, but the skin was intact. An upper GI study with barium showed images compatible with gastroptosis (Figure 1). During an upper digestive endoscopy, we noticed food residues in the stomach (Figure 2) and a narrow duodenal bulb, making it difficult to pass the endoscope through the duodenum. Initially, we thought of bulbar stenosis due to bulb ulceration. In this context, an antral biopsy was taken. The result was negative for helicobacter pylori, confirmed by a negative breath test. We treated her with a high dose of omeprazole for four weeks, but clinical signs were the same. In the context of growth failure, we excluded celiac disease and inflammatory bowel diseases (IBD). Serology for celiac disease and fecal calptrotectine were normal. An abdominal CT did not reveal an annular pancreas or other abnormalities compatible with IBD despite the view of an enlarged stomach. A second upper digestive endoscopy confirmed a post-bulbar stenosis approximately 4-5 mm. After many consultations, the decision was made that the definitive treatment would be surgery. During the intervention, it was noticed that the stomach was enlarged (Figure 3) which ended in a small and narrowed duodenum (Figure 4). Finally, it was clear that the cause of recurrent vomiting and abdominal pain was congenital stenosis of the duodenum, which age of the patient. The surgeons proceeded with an intervention similar to Billroth type 1 because of the enlarged stomach (Figure 5). After the surgical procedure, the girl became stable and did not present dysmotility symptoms. Gradually, she and was tolerating the liquid enteral nutrition and later solid foods. Initially, we used a transanastomotic tube to nourish her. She was discharged two weeks after the intervention. We performed another upper GI study with barium three months later, which showed a normal regular form and stomach position (Figure 6).

## 3. Discussion

Gastroptosis is a radiological finding. There are some mechanisms suggested to cause gastroptosis and GI symptoms. The downward displacement of the stomach is thought to be caused by the relaxation or stretching of the muscle or by a decrease in the muscle tone [7]. In the literature, gastroptosis seems to be associated with delayed gastric emptying [8, 9]. In our case, delayed gastric time was caused by congenital duodenal stenosis, which is very rare, making surgery the best option for recovery. Initially, because of patient's age, we thought of postbulbar stenosis due to a chronic bulbar ulcer. The misdiagnosis of postbulbar duodenal stenosis was made based on failure to examine the duodenum on gastroscopy and the interpretation of upper gastrointestinal barium examination showing a large stomach and obstruction beyond duodenal bulb. It is unexpected to discover a congenital abnormality of the duodenum in a 16-year-old. Despite gastrointestinal symptoms, her intellect was perfect. Our patient had no other diseases, including trisomy 21, congenital cardiac disease, and prematurity [10] associated with congenital duodenal obstruction. It has been reported that diagnosis of congenital duodenal obstruction (CDO) may be delayed even up to adolescence [11–13]. The delayed diagnosis in children may be explained because they can tolerate small feedings due to the incomplete nature of the obstruction. In some cases, duodenal stenosis goes unrecognized for long periods, leading to recurrent episodes of vomiting, failure to thrive, and aspiration pneumonia. In our case, the most frequent symptom was recurrent vomiting. Following surgery, infants frequently have a period of upper gastrointestinal dysmotility secondary to chronic in-utero obstruction of proximal duodenum and gastric dilatation. Several strategies are used to provide nutrition while normal gastrointestinal function returns. This includes intravenous (parenteral) feeding and enteral feeding into the bowel distal to the level of obstruction through a trans-anastomotic tube. No approach is shown to be superior to any other [14–16]. After taking into consideration the patient's age and enlarged stomach, our surgeons preferred a procedure similar to Billroth type 1. Postoperatively, the patient was started on enteral nutrition with a transanastomotic tube (TAT) combined with parenteral nutrition. The recovery period was uneventful, and the patient was discharged home two weeks after the intervention.

## 4. Conclusion

Gastroptosis is not diagnostic for a particular disease. This rare radiological finding in children may obscure uncommon diagnosis, such as congenital duodenal stenosis, which can present a diagnostic challenge beyond early childhood.

## Data Availability

All information is available in the clinical files archive of the University Hospital “Mother Teresa”, Tirana, Albania.

## Abbreviations

CDO: Congenital duodenal obstruction
TAT: Transanastomose tube
CT: Computed tomography
CD: Celiac disease
IBD: Inflammatory bowel disease
GI: Gastrointestinal.
